# Modulators of Nitric Oxide-Dependent Osteoinductive Activity of Human Red Blood Cells

**DOI:** 10.1055/a-1877-9870

**Published:** 2022-09-12

**Authors:** Maria Pavlaki, Kateryna Moiko, Adina Thomaidis, George Chalikias, Katrin Schäfer, Stavros Konstantinides, Dimitrios Tziakas

**Affiliations:** 1 Department of Cardiology, Democritus University of Thrace, Alexandroupolis, Greece; 2 Department of Cardiology, Cardiology I, University Medical Center of the Johannes Gutenberg University Mainz, Mainz, Germany; 3 Center for Thrombosis and Hemostasis, University Medical Center of the Johannes Gutenberg University, Mainz, Germany

To the Editor:

Recently, we reported on the role of red blood cells (RBCs) in vascular calcification. Specifically, we could show nitric oxide (NO)-dependent enhancement of calcification of vascular smooth muscle cells in vitro, and at sites of intramural and intravalvular microhemorrhage in vivo and ex vivo. 1 The endothelial isoform of NO synthase (eNOS) has been localized both in the cytoplasm and on the membranes of RBC 2 3 ; importantly however, we observed that the osteoinductive effects of intact RBC and those of RBC lysates were much less pronounced than those of RBC membranes. 1 To explain these differences, and to begin to understand their relevance for valvular and vascular diseases, we focused our attention on the role of intracellular factors and the local microenvironment in modulating NO bioavailability by affecting its generation and scavenging.

**Fig. 1:**
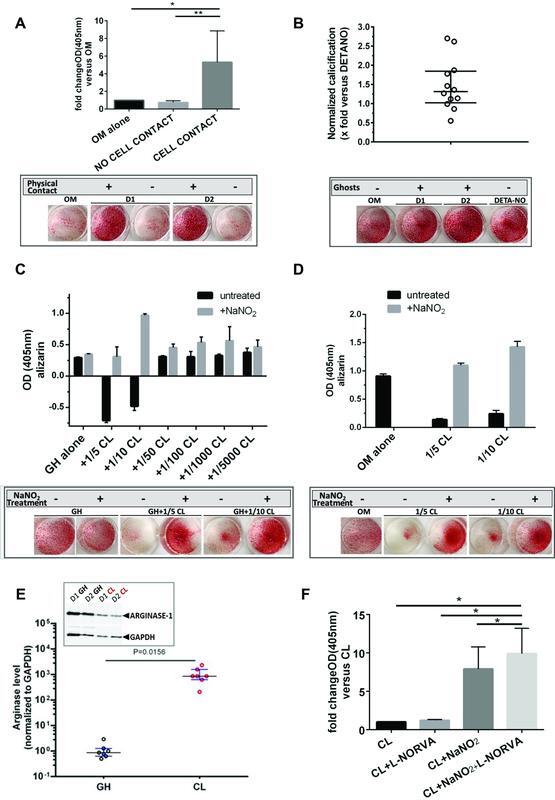
**Modulators of red blood cell osteoinductive activity.** ( **A** ) Ghost-dependent enhancement of calcification requires physical contact with target AoSMC; experiments using ghosts from *n* =3 donors in the AoSMC calcification assay. Ghosts were placed inside a transwell chamber with osteogenic medium (OM), either in direct contact or separated from AoSMC by a 0.4 micron pore filter. Following incubation for 5 days, cells at the bottom of the culture dishes were stained with alizarin red for visualization of calcific deposits. The upper part of the panel summarizes the values obtained with and without cell contact versus OM alone respectively, with OM value set as 1. Analyses were performed using paired *t* -test. Bars display mean values±SD of experiments performed twice. * *p* =0.049 for the difference between cell contact and OM alone, and ** *p* =0.015 for the difference between cell contact and no cell contact. Τhe lower part shows representative optical findings from two donors, D1 and D2. ( **B** ) Relative osteoinductive activity (alizarin index) of ghosts from healthy donors ( *n* =12). AoSMCs were treated with ghosts or 400 μΜ DETA-NONOate for 5 days under osteogenic conditions and stained with alizarin red. Extracted stain was measured photometrically (OD at 405nm). Following subtraction of the effect in the presence of OM only, OD values were normalized to the respective values obtained from the treatment with DETA-NONOate which was set as 1. Median and IQR are shown; median: 1.32, IQR: 1.02–1.85. Representative images from two donors are shown in the lower part of the panel. ( **C** ) RBC lysate inhibitory effect on ghost osteoinductive activity switches to enhancement following treatment with NaNO _2_ ( *n* =4 donors). Cell lysate (CL), added to the AoSMC assay together with a fixed amount of 5μg of membrane ghosts (GH), inhibited the osteoinductive activity of GH in a dose-dependent manner. The graph summarizes the effect of different amounts of CL combined with GH in one of the donors (D1). CL amounts are expressed as ratios (lowest: 1/5,000, highest: 1/5) of the quantity yielding 5μg of GH protein, the standard quantity applied in these experiments. Incubation of CL with NaNO _2_ converted Hb to metHb and fully reversed the inhibitory effect of CL on the osteoinductive activity of GH, a finding most prominent with CL quantities of 1/5 and 1/10. Displayed are mean values±SD obtained from two replicates for each condition per experiment, with experiments performed twice. Representative findings from the same donor are shown in the lower part of the panel. ( **D** ) CL-enhanced calcification of AoSMCs following treatment with NaNO _2_ ( *n* =4 donors). CLs were added to AoSMC in quantities as in the previous panel under osteogenic conditions (OM). The graph summarizes the effect of different amounts of CL combined with GH in one of the donors (D1). Displayed are mean values±SD obtained from two replicates for each condition per experiment, with experiments performed twice. Representative findings from the same donor are shown in the lower part of the panel. ( **E** ) Relative GH and CL arginase levels per (single) RBC differ by 1 order of magnitude (∼1,000×). Relative arginase content was measured in RBC from healthy donors ( *n* =7) by means of band densitometry of immunoblots of respective samples, normalized to the housekeeping GAPDH protein and also to the RBC number per sample run. Analyses were performed using the Wilcoxon matched-pairs signed rank test. Medians with IQR are shown. The insert shows a representative image of a Western blot with two samples. ( **F** ) Αrginase inhibition enhances the osteoinductive potential of RBC lysates, and these effects are additive to those of Hb oxidation. CLs from healthy donors ( *n* =4) were incubated with AoSMC under osteogenic conditions in the presence or absence of the arginase inhibitor L-norvaline (L-NORVA). Enhancement of osteoinductive activity in the presence of L-NORVA was observed only following conversion of oxyHb to metHb with NaNO _2_ . Means±SD are shown. * *p* <0.05 for the difference between CL+NaNO _2_ +L-NORVA and each one of the other conditions (CL, CL+L-NORVA, CL+NaNO _2_ ). AoSMCs, human aortic smooth muscle cell(s); CL, erythrocyte cell lysate(s); GH, (red blood cell membrane) ghosts; Hb, hemoglobin; IQR, interquartile range; NO, nitric oxide; OD, optical density; OM, osteogenic medium; RBC, red blood cell(s); SD, standard deviation.

Proximity between the source and the target is crucial for NO signaling due to the instability of the molecule, especially under the conditions of RBC extravasation during microhemorrhage into the vessel wall. To investigate whether a direct cell contact is required for calcification, RBC membranes (ghosts) were incubated with human aortic smooth muscle cells (AoSMC); ghosts were either separated from AoSMCs by transwell filters (0.4 μm) or placed on the cells directly, and the latter cultivated in the presence of an osteogenic medium (OM) using the in vitro calcification assay as previously described. 1 In this assay, calcification is assessed by alizarin staining of calcium phosphate deposits. As shown in the Fig. 1(A) , prevention of a direct contact between ghosts and AoSMC reduced ghost-mediated calcification to a level similar to the basal activity using OM alone. In further experiments ( Fig. 1B ), we demonstrated the variability of the osteoinductive potential of RBC membranes (ghosts) from healthy individuals ( *n* =12), using as a reference the effect of NO released by 400 μΜ DETA-NONOate (concentration chosen after a series of titration assays); this NO donor is characterized by slow release, which might better simulate the NO release by RBC ghosts in this specific assay.

In previous experiments, we compared distinct fractions of RBC and found that an amount of RBC lysates equivalent, in terms of protein content, to RBC ghosts did not induce calcification of AoSMCs; we also showed that the osteoinductive activity of ghosts was diminished when they were used together with an amount of heme corresponding to that isolated from the same RBC preparation. 1 These findings suggested a NO-scavenging role for oxygenated hemoglobin (OxyHb(Fe ^2+^ )) in the cytosol of RBC from collected blood. 4 5 OxyHb is preserved in this form by a functional redox regulation system in intact RBCs. 6 However, upon RBC extravasation and cell lysis, it is prone to oxidation to methemoglobin (metHb(Fe ^3+^ )) or other forms, 7 8 which are incapable of scavenging NO. 6 These reactions are promoted by the oxidative environment reported to exist under conditions of intraplaque hemorrhage. 7 8 9 10 11 Thus, to examine the changes of the osteoinductive potential of RBC lysates in dependence of the hemoglobin oxidation status, we tested the effect of RBC lysates isolated from blood, in which Hb exists in the oxyHb form, 12 13 against that of RBC lysates treated with NaNO _2_ , which converts oxyHb to metHb. 14 Conversion was visible as a color change from bright red to brown by visual observation and confirmed spectrophotometrically. 15 The osteoinductive effect on AoSMC by different amounts of RBC lysates (co-incubated with a standard amount of 5μg of ghost protein from the same donor) was compared with that of 5μg of ghost protein alone. In these experiments, RBC lysates corresponding to 1/5–1/10 of 5μg of ghost protein (based on the number of intact RBCs needed to be lysed to obtain the given ghost protein amount) exerted an inhibitory effect on calcification. This was in contrast to NaNO _2_ -treated lysates containing metHb, which enhanced the osteoinductive activity of ghosts. The results from four donors are summarized and representative results from one donor are shown in Fig. 1(C) . Of note, and as also shown in Fig. 1(C) , RBC lysate concentrations of 1/50 or less had no effect compared with untreated cells. Consistently, RBC lysates alone (in the absence of ghosts) exerted osteoinductive activity on AoSMC only after conversion of Hb to metHb with NaNO _2_ treatment ( *n* =4; representative findings from one donor displayed in Fig. 1D ). Statistical analysis comparing the osteoinductive effect of untreated with that of NaNO _2_ -treated lysates (focusing on the 1/10 concentration; paired *t* -test for four donors), yielded a *p* -value of 0.006 for the augmentation in the presence of NaNO _2_ when RBC lysates were combined with ghosts, and 0.001 when lysates were used alone.

Another factor that may interfere with NO availability is arginase, which competes with eNOS for their common substrate, L -arginine. 16 First, we quantitated the amount of arginase in ghosts and RBC lysates, and subsequently assessed the effect of arginase on osteoinductive activity using a specific inhibitor, L-norvaline, in lysates from the same donors. Using immunoblot band densitometry, the amount of arginase corresponding to a given number of RBC was 1,000-fold higher in RBC lysates compared with ghosts ( Fig. 1E) ). Inhibition of arginase by L-norvaline enhanced both the osteoinductive effect of RBC ghosts (confirming our previous results 1 ) and that of lysates ( Fig. 1F ). This effect varied, depending on the levels of arginase in the samples from different donors, which may explain, at least in part, the variability observed in Fig. 1(B) .

Taken together, our results suggest that both membrane and lysate fractions of RBCs exert NO-dependent osteoinductive activity, which requires a close contact of these components with target cells and is enhanced upon hemoglobin oxidation. Variability of NO bioavailability and RBC lysate-mediated calcification may be due to interindividual differences in the levels of arginase which is present in human erythrocytes and differentially regulated in cardiovascular disease states. 17
